# The burden of pediatric emergency departments, constipation: a systematic review

**DOI:** 10.1186/s43054-022-00101-6

**Published:** 2022-03-07

**Authors:** Emine Özdemir Kaçer, İlker Kaçer

**Affiliations:** 1 Department of Pediatrics, Aksaray University, Faculty of Medicine, 68100 Aksaray, Turkey; 2 Department of Emergency Medicine, Aksaray Training and Research Hospital, Taşpazar Mahallesi 859, Sokak No:23-A Park Vadi Evleri A Blok Daire: 16, 68100 Aksaray, Turkey

**Keywords:** Abdominal pain, Constipation, Dietary advice, Enema, Healthcare costs, Pediatric emergency department

## Abstract

**Background:**

Constipation constitutes an important part of emergency service applications in our country as well as all over the world. We aimed to illuminate the situation in our regional hospital regarding the increase in the emergency department density and the financial burden of applications due to constipation.

**Methods:**

This descriptive retrospective study was conducted in a regional tertiary hospital. The medical records of all constipation-related admissions to the emergency department between 01 January 2019 and 31 December 2019 were retrospectively reviewed. The etiology of constipation, complaints, physical examination, imaging studies, treatment modalities, and health expenses costs were recorded.

**Results:**

A total of 3271 patients aged 0–17 years (mean 4.24 ± 3.56 years) were included in the study. One thousand nine hundred and seventy-six (60.3%) of the patients were male, and 1295 (39.6%) of them were female. The majority of patients (*n* = 3107, 95%) were discharged without hospitalization. Health expenditure due to constipation was 834.626 Turkish liras. The health cost of the patients who were treated from the emergency room without hospitalization was 780.126 Turkish liras.

**Conclusions:**

In order to reduce unnecessary constipation applications and costs in emergency services, more detailed information should be given to prevent constipation during outpatient services, and dietary counseling should be provided when necessary.

## Background

Constipation is a common problem in pediatric patients. Diagnosis is made and followed up in pediatric emergency, pediatric outpatient clinics, and pediatric surgery outpatient clinics. In a study, it was found that abdomen X-ray used unnecessarily was very frequent in the management of constipation in pediatric emergency services, and the use of enemas can treat patients’ complaints in a short time [1]. In another study, it was determined that constipation is an important public health problem that affects the quality of life, and it also creates a significant financial burden for the pediatric emergency department [2].

Although our country’s health policy is very successful, the increase in emergency service applications and health expenses due to the increasing population of the social and technological developments causes the service provided to be inadequate. According to TUIK data, health expenditures increased by 21.7% compared to the previous year and reached 201 billion Turkish liras in 2019 [3]. This situation creates a great burden on both health workers and our country economically.

In this study, we aimed to illuminate the situation in our regional hospital regarding the increase in the emergency department density and the financial burden of applications due to constipation.

## Methods

### Study design

This retrospective descriptive study was carried out in the pediatric emergency department of a tertiary hospital in Aksaray, Turkey. In the period from 01 January 2019 to 31 December 2019, the medical records of patients admitted to the emergency department were retrospectively reviewed. The study was conducted in compliance with the Declaration of Helsinki and approved by Aksaray University School of Medicine, Aksaray Education and Research Hospital Scientific Research Evaluation Committee with decision no: 2021/03-16.

### Study population and setting

The following patients were included in the study: (1) under 18 years of age and (2) those diagnosed with constipation, abdominal pain, and encopresis in medical records for which history and physical examination suggest constipation in the emergency department. The following patients were excluded: (1) over 18 years old; (2) patients with previous abdominal surgery, with bile vomit, with abdominal distension, with rebound and tenderness, with abnormal vital signs, with fever, with the suppressed immune system, and with neurogenic bowel; and (3) missing data.

Gender (female, male), age, past illness history, physical examination findings, imagining, treatment, presenting complaints, hospitalization requirement, mortality, and healthcare costs were recorded.

### Statistical analysis

This is a clinical, retrospective, cross-sectional study. We used descriptive statistics and demographic information to define constipated patients. Student’s *t*-test for continuous variables and the chi-square test for categorical variables were used. All statistical analysis was performed using the SPSS software for Windows (SPSS Inc., Chicago, IL). A *P* value under 0.05 was considered statistically significant.

## Results

There were 54,342 total pediatric emergency departments admissions during the study period. A total of 6573 admissions were about abdominal pain. A total of 3633 admissions diagnosed as constipation were reviewed. A total of 292 patients who had missing data, 56 patients who were surgical, and 14 patients who had an additional clinical condition like cerebral palsy were excluded from the study. Finally, 3271 patients aged 0–17 years (mean 4.24 ± 3.56 years) including 1295 females (39.6%) and 1976 males (60.4%) were included in the study.

The most common presenting complaint was abdominal pain (*n* = 1760, 53.8%). X-ray was requested from 1325 (40.5%) children to diagnose the patients, and 1060 (32.4%) children were diagnosed with anamnesis and physical examination after no imaging examination was requested. Only the enema was applied to 1557 (47.6%) patients. Only intravenous (IV) hydration was applied to 775 (23.6%) patients (IV hydration means all intravenous administrations). A total of 199 patients have applied enema and intravenous hydration. Enema, laxative, and diet were recommended to all of the patients, while 1138 (34.8%) were discharged from the emergency department only with a prescription without any treatment. It was found that urinary tract infection was accompanied in 4% of the patients with constipation. The characteristics of the patients, physical examination findings, and diagnostic protocols are summarized in Table 1.

**Table 1:** The characteristics of the patients, physical examination findings, and diagnostic protocols

		Number	Percent
**Gender**	**Male**	1295	39.6
	**Female**	1976	60.4
**History**	**Painful bowel movements**	1813	55.4
	**Constipation**	999	30.5
	**Encopresis**	347	10.6
	**Anal fissure**	112	3.4
**Physical examination**	**Hard stool in the rectum**	1655	50.6
	**Abdominal mass consistent with stool**	1036	31.7
	**Suprapubic pain**	357	10.9
	**Encopresis**	177	5.4
	**Anal fissure**	46	1.4
**Imaging**	**None**	1060	32.4
	**X-ray**	1325	40.5
	**USG**	886	27.1
**Treatment**	**Enema**	1557	47.6
	**IV hydration**	775	23.6
	**Prescription**	1138	34.8
**Presenting complaints**	**Abdominal pain**	1760	53.8
	**Constipation**	571	17.5
	**Painful bowel movements**	269	8.2
	**Vomiting**	204	6.3
	**Bloody stool**	98	3
	**Urinary tract infection**	130	4
	**Encopresis**	177	5.4
	**Nausea**	31	0.9
	**Others**	31	0.9
**Readmission**	**Enema**	90	5.8
	**IV hydration or prescription**	351	20.4
**Result**	**Hospitalization**		
	**Pediatric service**	51	1.5
	**Pediatric surgery Service**	112	3.4
	**Discharged**	3107	95.1
	**Ex**	0	0
	**Transferred**	0	0

In total, 441 (13.4%) patients made repeated pediatric emergency applications. Of the re-admitted patients, 90 (5.8%) were from the enema-only group, and 351 (20.4%) were from the iv hydration and/or only prescription given group. All of the patients who applied again were consulted to the specialist physician. One hundred and sixty-three (5%) of the patients included in the study were hospitalized. Fifty-one patients (1.5%) were hospitalized to the pediatric service due to constipation, and 112 patients (3.4%) were hospitalized to the pediatric surgery service due to accompanying surgical problems. All of the hospitalized patients were hospitalized due to another concomitant disease. None of the patients was transferred to another hospital.

**Fig. 1:**
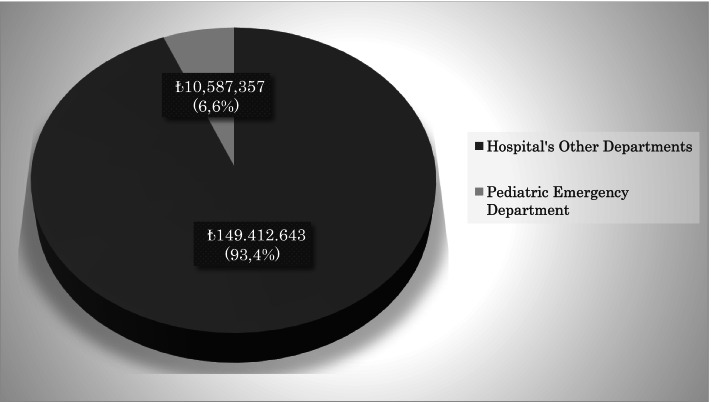
Annual healthcare costs of hospital

**Fig. 2:**
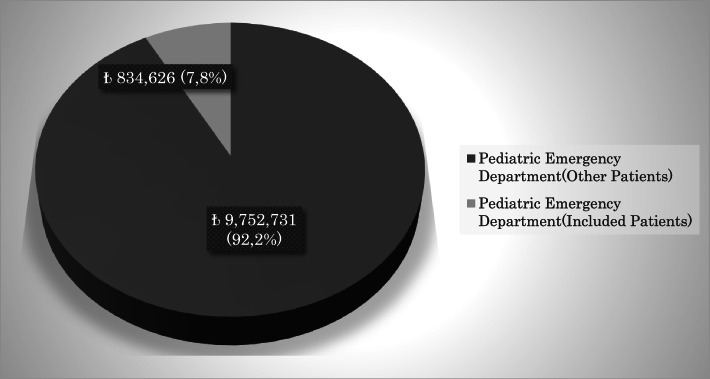
Healthcare costs of pediatric emergency department

**Fig. 3:**
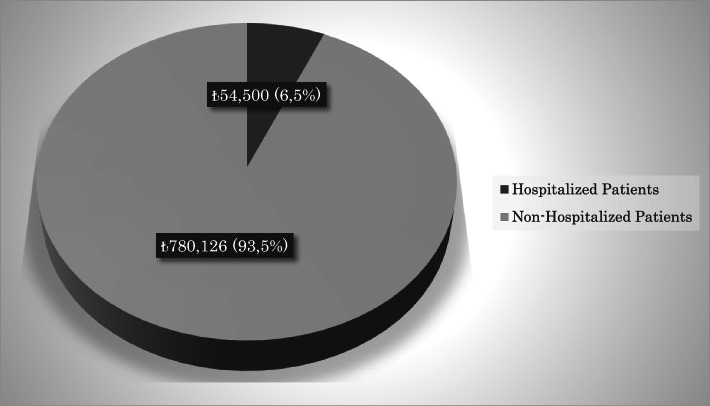
Healthcare costs of included patients

The total health service cost of our hospital during the study period was approximately ₺160,000,000 TL, and the cost of pediatric emergency service was 6.6% (₺10,587,357) of this cost. The total health care cost of the patients included in the study was ₺834,626. Non-hospitalized patients constituted 93.5% (₺780,126) of this cost, and hospitalized patients constituted 6.5% (₺54,500) of this cost. Hospitalized patients’ health cost was ₺160,000 for these patients during their stay in the hospital. The health service costs of our hospital are summarized in Figs. 1, 2, and 3.

## Discussion

In this study, we investigated the density in our regional hospital, the increase in the emergency department, and the financial burden of applications due to constipation. As a result of our study, the majority of applications due to constipation were discharged with various treatments and recommendations. The rate of patients requiring hospitalization was very low. Emergency service applications due to constipation increase both the intensity of the emergency service and the health costs. The use of laxatives reduces recurrent admissions to the emergency department due to constipation.

The lack of a generally accepted definition of constipation is one of the biggest clinical problems. There are some commonly used definitions (for example, Rome III, NASPGHAN, and PAACT), but all suggest different definitions [2, 4, 5]. The definition of constipation varies according to age groups [6]. Since these criteria are not used in emergency services, patients presenting with abdominal pain may be diagnosed with constipation at a high rate or incorrectly. For this reason, it would be more appropriate for constipated patients to use the outpatient clinic instead of the emergency room. In our study, we used history, complaint, and physical examination findings as the criteria for constipation.

Constipation accounts for more than half of abdominal pain. While the use of abdominal X-rays for the diagnosis of constipation in outpatient clinics is 5%, this rate rises to 70% in emergency services [7]. In our study, imaging was performed in 1325 (40.5%) of the patients who applied to the emergency department. This situation causes both a financial burden and unnecessary radiation exposure.

Abdominal X-ray usage is one of the most concerning finding in our study, and 1325 patients (40.5%) had abdominal X-rays. Our findings were lower than the literature [8, 9]. NASPGHAN recommends that the diagnosis has to be based on history and physical examination [4]. A systematic review indicated that abdominal X-ray has poor diagnostic accuracy [10]. On the other hand, Freedman et. al. suggested that using abdominal X-ray may mask underlying conditions [11]. They suggested that the presence of stool on abdominal X-rays does not rule out an alternative diagnosis. In our study, 37 patients (1.14%) were excluded from the study because they were acute appendicitis in addition to constipation. If the presence of stool seen on abdominal X-rays of these patients is not well evaluated, appendicitis would be masked.

Levy et al. found that one-third of children received enema [12]. Approaches of enema are found to be effective in the pediatric population [9]. Also, enema is thought to be discomfortable. According to Freedman et al., children treated with enema visit pediatric emergency departments more than those who do not receive an enema [11]. According to another study, enema and oral laxatives are equally effective for treating fecal impaction in the pediatric population [9]. Enemas relieve rectal pressure and have some impact on a stool throughout the rest of the gastrointestinal tract [8]. In our study, nearly half of the patients received an enema. While the readmission rate of emergency department in only the enema was applied patients was 5.8%, only given IV hydration and only given prescriptions patients’ readmission rate was 20.4%. It can be concluded that the use of laxatives reduces recurrent admissions to the emergency department due to constipation.

These days when the whole world is struggling with epidemics such as the COVID-19 pandemic and natural disasters such as fire and flood, it has become a necessity to use our financial resources and health facilities correctly. The health expenditure for the patients admitted to our pediatric emergency department due to constipation for a period of 1 year is ₺834.626. Although this amount seems small, it reaches a level that cannot be underestimated when the whole country is taken into account. This workforce and financial resource can use for the worse patients.

## Conclusion

We demonstrated that an important number of pediatric emergency department visits are related to constipation, which has to be managed in outpatient policlinics. We found that overutilization of abdominal X-rays and ultrasound is a burden for pediatric emergency departments. Long-term management and dietary counseling have to be explained to parents in detail. Further studies are needed to define constipation, and also to educate the parents.

### Limitations

Costs have also decreased as pediatric emergency service applications due to the COVID-19 pandemic have decreased by approximately 50% during the working period. This may cause our study data to appear less effective. Our study is limited by the data available in the patient medical records. The entire patient population could not be reached due to the lack of a precise definition of constipation. The rate of enema use or dieting in prescribed patients is unknown.

## Data Availability

None declared

## References

[1] Patel H, Law A, Gouin S (2000). Predictive factors for short-term symptom persistence in children after emergency department evaluation for constipation. Arch Pediatr Adolesc Med.

[2] Mugie SM, Benninga MA, Di Lorenzo C (2011). Epidemiology of constipation in children and adults: a systematic review. Best Pract Res Clin Gastroenterol.

[3] Institute TS (2019). Health Spending Statistics.

[4] Bulloch B, Tenenbein M (2002). Constipation: diagnosis and management in the pediatric emergency department. Pediatr Emerg Care.

[5] Lewis LG, Rudolph CD (1997). Practical approach to defecation disorders in children. Pediatric Ann.

[6] Diamanti A, Bracci F, Reale A, Crisogianni M, Pisani M, Castro M (2010). Incidence, clinical presentation. Am J Emerg Med.

[7] Kearney R, Edwards T, Bradford M, Klein E (2019). Emergency provider use of plain radiographs in the evaluation of pediatric constipation. Pediatr Emerg Care.

[8] Liem O, Harman J, Benninga M, Kelleher K, Mousa H, Di Lorenzo C (2009). Health utilization and cost impact of childhood constipation in the United States. J Pediatr.

[9] Russo M, Strisciuglio C, Scarpato E, Bruzzese D, Casertano M, Staiano A (2019). Functional chronic constipation: Rome III criteria versus Rome IV criteria. J Neurogastroenterol Motil.

[10] Van Den Berg MM, Benninga M, Di Lorenzo C (2006). Epidemiology of childhood constipation: a systematic review. Am J Gastroenterol.

[11] Freedman SB, Thull-Freedman J, Manson D, Rowe MF, Rumantir M, Eltorki M, Schuh S (2014). Pediatric abdominal radiograph use, constipation, and significant misdiagnoses. J Pediatr.

[12] Levy EI, Lemmens R, Vandenplas Y, Devreker T (2017). Functional constipation in children: challenges and solutions. Pediatr Health Med Therapeut.

